# Oral Cholera Vaccine Efficacy and Effectiveness

**DOI:** 10.3390/vaccines9121482

**Published:** 2021-12-15

**Authors:** Katerina Rok Song, Jacqueline Kyungah Lim, Se Eun Park, Tarun Saluja, Sung-Il Cho, Tram Anh Wartel, Julia Lynch

**Affiliations:** 1 International Vaccine Institute, Seoul 08826, Korea; katerina.song@ivi.int (K.R.S.); kalim@ivi.int (J.K.L.); seeun.park@ivi.int (S.E.P.); tarun.saluja@ivi.int (T.S.); anh.wartel@ivi.int (T.A.W.); 2 Graduate School of Public Health, Seoul National University, Seoul 08826, Korea; scho@snu.ac.kr; 3 Graduate School of Public Health, Yonsei University, Seoul 03722, Korea

**Keywords:** vaccine efficacy, vaccine effectiveness, oral cholera vaccine (OCV), cholera, prevention, vaccine

## Abstract

Although measuring vaccine efficacy through the conventional phase III study design, randomized, double-blinded controlled trial serves as the “gold standard”, effectiveness studies, conducted in the context of a public health program, seek to broaden the understanding of the impact of a vaccine in a real world setting including both individual and population level impacts. Cholera is an acute diarrheal infection caused by the ingestion of food or water contaminated with the bacterium *Vibrio cholerae*. Since the 1980s, either killed or live oral cholera vaccines (OCVs) have been developed and efficacy and effectiveness studies have been conducted on OCV. Although the results of OCV effectiveness studies sometimes showed outliers, the tendency seen is for effectiveness of the vaccine used in public health settings to be somewhat higher than estimated in randomized controlled trials due to the influence of indirect herd protection. Efficacy and Effectiveness studies both generate important information about the vaccine performance characteristics and its impact when used in real world populations at risk for the disease.

## 1. Introduction

### 1.1. Vaccine Efficacy and Effectiveness

Vaccine efficacy and effectiveness are generally calculated using a similar mathematical formula that is 1 minus some measure of relative risk (RR) in the vaccinated group compared with the unvaccinated group: Percentage vaccine efficacy/effectiveness = (1 − RR) × 100 [1]. Measuring vaccine efficacy through the conventional design of phase III randomized, double-blind, controlled clinical trial serves as the “gold standard” method for evaluating vaccines in a way that both preserves the rights of human participants and safeguards against bias [2]. Despite these strengths, efficacy trials which intentionally select ideal subjects (excluding individuals who cause variation), administer vaccine in highly structured settings, and rigorously follow subjects and outcomes can be imperfect in predicting the performance of a vaccine when it is implemented among a diverse population in routine public health practice [3]. Efficacy trials are primarily designed to measure the direct effect of a vaccine on those who receive it at the individual level, but vaccination induces various kinds of effects, both at the individual and population level [4]. Population-level vaccine effectiveness can be further categorized into the ‘direct’, ‘indirect’, ‘total’, and ‘overall’ impact of the vaccine [5]. Thus, effectiveness studies, typically conducted in the context of a public health program, seek to broaden the understanding of the impact of a vaccine in a real world setting including both individual and population level impacts. The conventional sequence of studies of new vaccines defers the evaluation of effectiveness until phase IV, after licensure [3,6].

Oral Cholera vaccine (OCV) development was initiated in the 1980s at the University of Gothenburg in Sweden using killed whole-cell bacteria [7]. With a vaccine development history of nearly 50 years, both efficacy and effectiveness data on OCV have accumulated. Through this article, we would like to review this history and understand how efficacy and effectiveness studies contributed to the implementation of OCV as a public health tool.

### 1.2. Cholera

Cholera is an acute diarrheal infection caused by ingestion of food or water contaminated with the bacterium *Vibrio cholerae*. A non-invasive organism, ingestion can be asymptomatic or cause acute watery diarrhea ranging from mild to profoundly severe principally through elaboration of a toxin. Cholera remains a global threat to public health and an indicator of inequity and lack of social development. Researchers have estimated that every year, there are roughly 1.3 to 4.0 million cases, and 21,000 to 143,000 deaths worldwide due to cholera [8].

*Vibrio cholerae* is a Gram-negative, highly motile curved rod shaped bacteria with a single polar flagellum. *V. cholerae* is classified by the composition of its major surface antigen (O) from lipopolysaccharide into nearly 206 serogroups. Only two serogroups of *V. cholerae*, O1 and O139, are considered causative agents of epidemic cholera [9]. *V. cholerae* O1 has two biotypes, classical and El Tor. Each biotype has three serotypes: Ogawa, Inaba, and Hikojima. *V. cholerae* O1 Hikojima is an unstable form and rarely occurs in nature [10]. The current seventh pandemic of cholera came as a result of the emergence of the El Tor strain, which is more adapted and persistent in the environment, and may cause higher infection to case ratio with more asymptomatic carriers than the classical counterpart [11,12].

### 1.3. Cholera Vaccines

The first vaccine against cholera, a whole-cell (WC) injectable vaccine, was developed in 1885. Although several additional injectable cholera vaccines were developed in the late 19th and early 20th century, they were shown to be reactogenic with limited efficacy and unsuitable for large scale public health programs. Vaccination against cholera was eventually removed by the World Health Organization (WHO) from recommended cholera-control measures in 1973 [13]. Since the 1980s, based on the belief that immune protection against cholera primarily depends on stimulation of local mucosal immunity in the intestine, OCVs comprised of either killed or live cells have been developed and made commercially available.

## 2. Killed Whole-Cell Monovalent (O1) Vaccine with Cholera Toxin B Subunit (WC-CTB): Dukoral^®^

Dukoral^®^, initially produced by SBL Vaccine and now produced by Valneva, was the first registered and WHO pre-qualified OCV. The product contains a total of 1.25 × 10^11^ bacteria with equal parts of four different *V. cholera* strains (Table 1). It is the only OCV that also contains 1 mg of recombinant cholera toxin B subunit (rCTB). Because of the recombinant protein, the vaccine suspension must be administered with an antacid to prevent the destruction of the protein in the stomach [14].

Therefore, each dose of vaccine is supplied as one vial of suspension (containing the inactivated bacteria and rCTB) together with one sachet of effervescent granules (sodium hydrogen carbonate) which is dissolved in an age dependent volume of water and mixed with the suspension. 

Early studies evaluated several formulations varying in strain composition. In most early studies, formulations with CTB (often denoted as Whole Cell-B subunit or WC-BS), and without the CTB (referred to as WC) were directly compared.

In early formulations of WC-BS, the CTB component was produced through purification of cholera toxin isolated from culture, and subsequently, it was produced using recombinant technology (rCTB) [15].

Challenge studies in human volunteers provided the first demonstration of efficacy. The challenge study conducted at the University of Maryland enrolled healthy participants aged 19–35 years. Participants receiving either WC-BS (with 5 mg of CTB) or WC were given three doses at 2-week intervals. Cimetidine was administered 3 h prior to receipt of vaccine in addition to the sodium bicarbonate solution mixed with the vaccine. Vaccinated participants and unvaccinated controls were challenged with 2 × 10^6^ El Tor Inaba *V. cholerae* (strain N16961) four weeks after completion of the third dose of WC (*n* = 9), and five weeks after completion of WC-BS (*n* = 11). The vaccine efficacy of WC was found to be 56%, and for WC-BS, 64% (Table 2). Among vaccinated participants in both groups that developed cholera, they had less severe illness compared to controls and complete protection from severe diarrhea [16].

With this preliminary demonstration of efficacy, both WC and WC-BS were evaluated in a large randomized, controlled field trial in Matlab, Bangladesh beginning in 1985. Of note, the quantity of CTB in this trial and subsequent formulations was reduced to 1 mg.

The entire population of Matlab at the time was estimated as 190,000, but trial eligibility was limited to the approximately 124,035 persons who were either children 2–15 years of age or women older than 15 years. Individuals were offered enrollment and randomized to one of three arms: WC-BS, WC or K12, a placebo composed of heat inactivated whole *Escherichia coli* in quantities sufficient to create a liquid with turbidity similar to WC-BS and WC. Participants received three doses of one of these formulations at approximately 6 week intervals. Approximately 89,596 persons received at least one dose of a study agent and 63,498 were vaccinated per protocol. Surveillance for diarrhea was maintained at all three treatment centers in Matlab. Efficacy was assessed at 4 timepoints (6 months, 1 year, 3 years, and 5 years) over the 5-year follow-up period and reported for two age groups (2–5 year, >5 years) [17,18,19,20]. During the period of surveillance, cases of Classical Ogawa, El Tor Inaba, and Classical Inaba were all isolated in substantial proportions in the trial area.

At the six month interval, the estimated efficacy for WC-BS was 85% (95% CI: 62–94, *p* < 0.0001) and consistent across age groups [17]. The efficacy of WC was 58% (95% CI: 14–79, *p* < 0.01). However, at the one year interval, the cumulative efficacy for WC-BS had dropped to 62% (lower bound 47%, *p* < 0.0001) with the estimated efficacy for those age 2–5 years only 38% (*p* < 0.05) (Table 2) [18]. The efficacy for the WC formulation was sustained at 53% (lower bound 34%, *p* < 0.001), but also with a declining efficacy in the youngest children. At the completion of the three-year follow-up the cumulative efficacy was 50% for WC-BS and 52% for WC, but in the third year there was no evidence of any protection for children 2–5 years of age with either product [19]. The efficacy for WC-BS was significantly greater than for WC only during the initial 8 months of follow-up. Because both biotypes were in circulation in Matlab, investigators evaluated the relative efficacy and found that the vaccine efficacy was higher against classical cholera (58% for WC-BS; 60% for WC) than against El Tor cholera (39% and 40%). Of note, exploratory sub-analysis found that the efficacy of two doses was not inferior to three doses [19]. Subsequent immunogenicity studies indicated that two doses of WC-BS administered two weeks apart produced an immune response equivalent to three doses given six weeks apart [21,22]. Thus, alternative two-dose regimens were explored in subsequent trials.

One of the distinguishing features of the CTB containing OCVs was first identified in a later analysis of the Bangladesh trial data. Individuals completing at least two doses of WC-BS, (but not WC) had a 67% (*p* < 0.01) protection against all episodes of heat labile enterotoxin producing *Escherichia coli* (LT-ETEC) diarrhea, and 86% (*p* < 0.05) protection against severe LT-ETEC diarrhea during the first three months of follow-up [23]. The hypothesis for this cross protection is the structural similarity between the B subunit of cholera toxin and the heat labile enterotoxin (LT) of *Escherichia coli*. As notable as this level of protection was, there was no indication of protection beyond three months. This finding of cross protection to LT-ETEC was confirmed in a subsequent randomized, controlled trial of WC-BS among short term tourists traveling to Morocco [24,25,26].

The field study in Bangladesh raised the possibility of several shortcomings of the WC-BS and WC vaccines. Specifically, that the formulation provided less protection against El Tor strains and among individuals with an O blood type. With El Tor cholera emergent in South America in the early 1990s amid a population with predominantly O blood type, two efficacy trials were concurrently undertaken in Peru with strikingly different outcomes.

The first was a randomized controlled trial enrolling 1,563 recruits of the Peruvian military from three military training centers near Lima, Peru between January and March 1994. This was the first trial to use the recombinant CTB in the formulation (WC-rBS) and compare to K12 placebo control both administered as two doses 7–14 days apart upon arrival at the training site. Passive surveillance was conducted for 18 weeks at the recruit medical clinics and during this time the training centers experienced a higher than expected attack rate. El Tor Ogawa was the dominant circulating strain and the protective efficacy against symptomatic cholera was 86% (95% CI: 37–97, *p* < 0.01) (Table 2). This trial demonstrated onset of protection within two weeks of vaccination using an accelerated and abbreviated regimen (two doses administered within 14 days) among a population presumed to be largely immune naïve and predominantly blood group O in a high transmission setting.

In contrast, a community-based study was conducted in Pampas, Peru on the outskirts of Lima. Among an estimated 39,725 eligible participants aged 2–65 years, 17,799 were recruited from the trial area and randomized to receive two doses of either WC-rBS or K12 two weeks part, followed by a booster 10 months later (14,997 participants) just prior to the second cholera season. There was no significant difference in gender, mean age (19 years), locale, or socioeconomic index between vaccine and placebo recipients. During the first cholera season, the estimated vaccine efficacy was −4% (95% CI: −88–43, *p* = 0.92) with isolation of *V. cholera* O1 among 17 vaccinees and 16 placebo recipients with 90% of the isolates being El Tor Ogawa [27]. During the second cholera season and following the booster dose at 10 months, the efficacy was 61% (95% CI: 28–79, *p* = 0.004) and 82% (95% CI: 28–89, *p* = 0.01) for severe cholera requiring hospitalization. In a small adult immunogenicity subset, among those vaccinated (*n* = 57) the seroconversion rate and GMTs were higher for Inaba than Ogawa, in fact only 16% of participants achieved a 2-fold increase in Ogawa vibriocidal titers after two doses. In the Bangladesh field trial, immune responses were poorer among young children than adults. The authors hypothesized that the disappointing results of the two-dose regimen in this community-based study as compared to the Peruvian military may be related to the younger age distribution of participants exaggerating further the suboptimal immune response to the Ogawa strain. However, the study was also criticized as potentially flawed in design and implementation of surveillance for cholera thus yielding falsely negative results in the first year [28].

WC-rBS became registered for use under the trade name Dukoral^®^ in 1991 with the combined indication for the prevention of and protection against cholera and ETEC producing heat-labile enterotoxin (LT) among adults and children 2 years and older visiting locations with risk of exposure to these pathogens. For adults and children older than 6 years, 2 oral doses with buffer are to be taken at least 1 week apart. For the primary immunization of children 2–6 years, 3 oral doses at least 1 week apart are recommended.

Following registration and at the request of the WHO, a pilot vaccination campaign deployed Dukoral^®^ pre-emptively in high risk refugee settlements in Uganda in 1997 in order to assess the feasibility and acceptability of the vaccine administered as a two-dose regimen [29]. The campaign achieved 83% coverage of at least one dose in the targeted communities and when a cholera outbreak occurred in the district the following year, there were no recorded cases of cholera in the vaccinated communities in spite of high attack rates in the surrounding local villages [30,31]. Although not a formal study, this real-world evidence of vaccine impact together with the clinical trial evidence led an expert group convened by the WHO to encourage additional pilot studies and consideration for pre-emptive use in high risk settings.

A field effectiveness study was designed in an endemic area with frequent outbreaks in Beira, Mozambique in 2003. Among the 21,818 persons in the targeted community, it was estimated that 19,550 were two years or older, non-pregnant, and eligible for vaccination, with 14,164 (72%) receiving a complete first dose, and 11,070 (57%) receiving both doses with a minimum of 15 days between. Surveillance was established at the community Cholera Treatment Centers and effectiveness was assessed using a case control study enrolling 43 persons with cholera diarrhea and 172 gender and age matched neighborhood controls without diarrheal illness. In addition, a bias indicator case-control study was used to detect bias by comparing non-cholera diarrhea and controls without diarrhea in the same population. During the five-month surveillance period, El Tor Ogawa was the predominant circulating type. The estimated effectiveness for those completing the two dose regimen was 84% (95% CI: 43–95 *p* = 0.005), and 78% for one or more doses (95% CI: 39–92, *p* = 0.004) after adjustment for potentially confounding variables (Table 2) [32]. Protection was not diminished in those under five years, nor in those older than 15 years where the HIV prevalence was presumed high (20–30%). The bias indicator study suggested the results of the cholera effectiveness could not be attributed to bias as there was no impact of vaccination upon non-cholera diarrhea.

Because this effectiveness study was conducted in a real-world setting, it also yielded data regarding the feasibility and cost of delivery of this vaccine through mass vaccination. The delivery costs and effectiveness data contributed to one of the early publications demonstrating cost effectiveness of the oral cholera vaccine [33,34].

Another case-control and bias indicator study was conducted in Zanzibar in 2009 where three peri-urban and three rural communities with endemic cholera were targeted for a mass vaccination campaign. This study extended the traditional assessment of effectiveness at protecting the individuals who received vaccine to include the indirect protection of neighbors that did not receive vaccine. A census completed prior to the vaccination campaign Geographic Information System (GIS) mapped all households in the targeted communities. Out of an estimated 48,178 eligible persons (>2 years and non-pregnant), 23,921 completed the two-dose regimen achieving an overall 50% vaccine coverage, however the actual coverage rate varied between neighborhoods. Over the 15-month surveillance period, the direct effectiveness of the vaccine was estimated as 79% (95% CI: 47–92, *p* < 0.0001) (Table 2). Indirect or herd protection was demonstrated by a decrease in the risk for cholera among non-vaccinated residents within a neighborhood as the vaccine coverage in that neighborhood increased [35]. The bias indicator study found no evidence of bias in the estimation of either direct or indirect effectiveness. Similar to Beira, conduct of the study in this real-world setting yielded additional important information needed to inform public health programs regarding feasibility and optimization of mass vaccination campaigns, and cost effectiveness [36,37]. At the time of this vaccination campaign and as a result of lack of data from clinical trials, recommendations did not yet support the vaccination of pregnant women even though they are at substantially higher risk of morbidity and mortality when infected with cholera. Consequently, pregnant women were by policy excluded from participation in the vaccination campaign. However, a follow-on observational study in the population found that 196 women who had received vaccine were in early pregnancy. A retrospective analysis found no statistically significant differences in the odds ratios for adverse birth outcomes among the exposed and unexposed pregnancies [38]. This early reassuring observational data regarding gestational effects of exposure to OCVs contributed to the eventual policy endorsement of including pregnant women in vaccination campaigns.

With the exception of the one study in Pampas, Peru, Dukoral^®^ demonstrated high levels of efficacy and effectiveness (Table 2); however, effectiveness studies provided considerable additional utility with observations in special populations including safety in pregnant women, and safety and effectiveness in a population with high HIV seropositivity, all individuals typically excluded from clinical trials. In addition, study of the vaccine in real-world settings provided valuable information with regard to indirect protection of vaccinated neighbors, feasibility and acceptability of use in public health programs, and cost utility.

Despite the availability of this WHO-prequalified cholera vaccine, OCV demand remained low. The main reasons for low demand were probably the price (one dose of Dukoral^®^ costs about $6 to the public sector), and the requirement of co-administration with buffer as the vaccine contains a rCTB sensitive to the acidic environment in the stomach [7]. In order to achieve a vaccine with lower cost and easier delivery, the further development of the WC vaccine was pursued.

## 3. Killed Whole-Cell Bivalent (O1 and O139) Vaccines: ORC-Vax^TM^/mORCVAX^TM^, Shanchol^TM^, Euvichol^®^/Euvichol-Plus

### 3.1. ORC-Vax^TM^

The University of Gothenburg transferred its WC technology to VaBiotech, a vaccine manufacturer under the Ministry of Health of Vietnam, an OCV without CTB and with a slightly different strain composition containing a classical Inaba strain 569B which efficiently expressed a putatively protective fimbrial antigen, toxin-coregulated pilus [39,40]. In a 1992 open field trial involving 119,033 participants in the city of Hue, Vietnam, the protective efficacy of this vaccine was shown to be 66% (95% CI: 46–79) for all ages after two doses, with similar results in children aged 1–5 years and adults during an outbreak of El Tor Ogawa cholera 8–10 months after vaccination [39].

In the early 1990s, *V. cholerae* O139 emerged in Bangladesh and India and the vaccine was reformulated into a bivalent vaccine with O139 added for protection against the new strain, and licensed as ORC-Vax™ in 1997 [41]. In 1998, a demonstration project was initiated in Hue city to assess the impact of vaccination with ORC-Vax™ under programmatic conditions. Half of the city’s communes were randomly selected for two-dose mass oral cholera vaccination. No cholera was observed during the 2 years of intensive surveillance after 1998. The remaining communes were immunized in 2000 with a modified vaccine which no longer included the Cairo 48 strain (Table 1). No cholera was observed in Hue until 2003 when a major outbreak of cholera occurred, and long-term protection was able to be measured associated with the two earlier mass immunization campaigns. A case-control study was conducted and the overall vaccine effectiveness 3–5 years after vaccination was 50% (95% CI: 9–63%) [42].

In October 2007, an increase in acute watery diarrhea cases was reported in Hanoi, caused by a genetically altered *V. cholerae* O1 Ogawa biotype El Tor producing classical biotype cholera toxin which had not previously been isolated in Vietnam. A vaccination campaign was conducted in two districts of Hanoi in 2008 [43]. Residents of four districts of Hanoi admitted to one of five hospitals for acute diarrhea were recruited for a matched, hospital-based, case-control effectiveness study. The vaccine was 76% protective against cholera in this setting (95% CI: 5–94, *p* = 0.042) after adjusting for intake of dog meat or raw vegetables, and consumption of boiled or bottled water most of the time [43].

To ensure the vaccine met international Good Manufacturing Practice (GMP) standards and WHO production guidelines for global use, the International Vaccine Institute (IVI) (Seoul, Republic of Korea) partnered with VaBiotech (Hanoi, Vietnam) to again reformulate the vaccine and express the antigen content in ELISA units (Table 1). This new formulation became registered in Vietnam as mORC-Vax™ and has been used extensively in that country to eliminate cholera. For the reformulated vaccine to be made available internationally, IVI facilitated a technology transfer between VaBiotech and Shantha Biotechnics Ltd., a private biotech company in Hyderabad, India (acquired by Sanofi Pasteur in 2009), a country with a national regulatory authority certified as fully functional by WHO.

### 3.2. Shanchol^TM^

Shanchol^TM^ is a killed WC vaccine consisting of killed whole-cell O1 and O139 serogroups without B subunit cholera toxin, manufactured by Shantha Biotechnics Ltd. (acquired by Sanofi Pasteur). It is a two dose oral vaccine to be taken with a minimum interval of two weeks; immunity against cholera is expected to appear 7–10 days after the second dose [44].

#### 3.2.1. Efficacy

The cluster-randomized placebo-controlled efficacy trial to evaluate the efficacy of Shanchol^TM^ was conducted in Kolkata, India in 2006. Cholera is endemic in this part of Kolkata and has a distinct seasonality. Clusters used in randomization were dwellings, which were randomly assigned to receive either vaccine or placebo, so that individuals living in the same dwelling (cluster) received the same intervention. A dwelling was defined as a hut, a group of huts, or a multistorey building with several households sharing water pipes, bathrooms, and latrines as assigned by the Kolkata Municipal Corporation [45]. The primary objective of the study was to evaluate the vaccine efficacy of a two dose regimen with a two-week interval. The primary analysis included 1721 clusters with 31,932 two dose vaccine recipients and 1757 clusters with 34,968 two dose placebo recipients [45]. During two years of follow-up, there were 20 episodes of cholera in the vaccine group and 68 episodes in the placebo group showing a protective efficacy of 67% (one-tailed 99% CI, lower bound 35%, *p* < 0.0001) [45]. The cumulative protective efficacy of Shanchol^TM^ at five years was 65% (95% CI: 52–74, *p* < 0.0001) (Table 3). Differences were observed between age groups: 1 to 4 years 42% (95% CI: 5–64); 5 to 15 years 68% (95% CI: 42–82); 15 and above years 74% (95% CI: 58–84) [46]. In 2009, Shanchol^TM^ was registered in India and in 2011 WHO Prequalification Program approved the vaccine, making it the first affordable OCV ($1.85 per dose) available for the global public market [7].

In order to explore the potential value of a regimen easier to use in an outbreak setting, a single dose OCV study was conducted in Dhaka, Bangladesh in 2013. Participants were randomly assigned to receive either vaccine (102,552 participants) or placebo (102,148 participants), of whom 204,700 were included in the per-protocol analysis [47]. During two years of follow up, adjusted overall protective efficacy for initial cholera episodes was 39% (95% CI: 23–52) and 50% (95% CI: 29–65) for severe cholera (Table 3). In participants aged 5 to 14 years, vaccine efficacy was 52% (95% CI: 8–75) against all cholera episodes and 71% (95% CI: 27–88) against severe cholera episodes. For participants aged 15 years or older, vaccine efficacy was 59% (95% CI: 42–71) against all cholera episodes and 59% (95% CI: 35–74) against severe cholera. The protection in the older age groups was sustained throughout the two year follow-up. However, among those younger than 5 years, the vaccine did not show protection against either all cholera episodes (−13% (95% CI: −68–25)) or severe cholera episodes (−44% (95% CI: −220–35)) [48,49]. This study did not result in a label change for the dosing regimen; however, the substantial protection among older children and adults did embolden others to explore the effectiveness of the single dose regimen in austere outbreak settings where providing the second dose two weeks after the primary dose was impractical.

#### 3.2.2. Effectiveness

In 2013, Gavi established a global stockpile of OCV to encourage use through mass vaccination campaigns in outbreaks and eventually in preventive campaigns in communities at risk for an outbreak [50]. As a result of vaccine availability, several effectiveness studies of Shanchol^TM^ have been conducted in different countries and settings. Effectiveness studies in an outbreak context have occurred in Haiti, Guinea, Malawi, Sudan, and Zambia, with the latter two exploring short term effectiveness of the single dose regimen. Other studies carried out in India and Bangladesh estimated effectiveness as a preventive intervention in a high-risk setting.

##### Effectiveness Studies in Outbreak Setting

In 2010, Haiti experienced a severe and prolonged cholera outbreak following re-introduction of the bacteria. Between April and June 2012, the first reactive OCV campaign in response to the cholera outbreak was conducted using the WHO-prequalified OCV Shanchol^TM^ in two sites in Haiti; an urban slum in Port-au-Prince and rural Artibonite valley [51]. A total of 97,774 people living in these sites were vaccinated, including 52,357 people living in Port-au-Prince (approximately 75% of 70,000 inhabitants) and 45,417 people in the Artibonite valley, with 91% receiving two doses [51,52]. A second campaign took place in Mirebalais in the Central Department from August to September 2014. Both campaigns aimed to deliver two doses of OCV 2 weeks apart [53].

A post-vaccination follow-up study was conducted between 2012 and 2015 to estimate the effectiveness of reactive oral cholera vaccination in the urban slum site of Port-au-Prince [52]. The stool culture-confirmed cases of cholera admitted to the Groupe Haïtien d’Etude du Sarcome de Kaposi et des Infections Opportunistes (GHESKIO) cholera treatment center was evaluated for vaccination status and area of residence during the 37 months follow-up period post-vaccination [52]. Of 1788 patients with culture-confirmed cholera, 1770 (99%) were either from outside the vaccine area (1400 cases) or from the vaccinated community but had not received OCV (370 cases). Of the 388 people from the catchment area who developed culture-confirmed cholera, 370 occurred among the 17,643 people who had not been vaccinated (2.1%) and the remaining 18 occurred among the 52,357 people (0.034%) who had been vaccinated (*p* < 0.001), for an effectiveness that approximates 97.5% (Table 4) [52]. Overall, the authors demonstrated the sustained impact of Shanchol^TM^ over three years post first reactive vaccination campaign performed in an urban slum of Haiti [52].

Another case-control study was conducted between 2012 and 2014 to measure vaccine effectiveness in the rural Artibonite Department. An estimated 76.7–92.7% of the Bocozel community, and 62.5% of the Grand Saline community were vaccinated against cholera in the 2012 campaign. Acute watery diarrhea patients seeking treatment at three cholera treatment units were tested by a rapid test and stool culture for *V. Cholerae* [51]. Among the 114 eligible individuals who presented with acute watery diarrhea, 47 cases with stool culture positive results were included in analyses of vaccine effectiveness in the case-control study. For each cholera case, four community-based people were recruited from their residences and assigned to the control group. In multivariable analyses, the vaccine efficacy was 63% (95% CI: 8–85) by self-reported vaccination, and 58% (95% CI: 13–80) for verified vaccination (Table 4). Vaccine effectiveness for individuals less than 5 years old was 50% (95% CI: −850–97, *p* = 0.70) and 72% (95% CI: 36–88) for those 5 years old and above. The vaccine was shown to be effective in protecting against cholera during the study period from 4 through 24 months after vaccination [51].

For long-term effectiveness assessment of the vaccine, the study was continued through 2016 with an addition of participants from the second campaign in the Central Department in 2014. Nevertheless, participants from the Artibonite Department, with their earlier campaign, contributed the majority of study information on vaccine effectiveness beyond 24 months. The vaccine effectiveness of one and two doses of Shanchol^TM^ was measured through an extended case-control study [53]. 178 people were assigned to the cholera case group and 706 to the control group. The study result showed that two dose effectiveness did not decrease during follow-up. In the adjusted analyses, the average cumulative 4 year effectiveness for two doses was 76% (95% CI: 59–86) for all ages, 77% (95% CI: 58–88) for those 5 years old and above. In contrast, single dose effectiveness decreased over time in a log-linear fashion, showing 79% of vaccine effectiveness at the end of 12 months (95% CI: 43–93), which declined to zero before the end of the 24 months. Unadjusted one dose vaccine effectiveness was 10% in children aged less than 5 years (95% CI: −468–86), and two dose vaccine effectiveness was 32% (95% CI: –117 to 79) [53].

A cholera outbreak was declared in Guinea in February 2012. A matched case-control study was conducted between May and October 2012 to evaluate the short-term effectiveness of two doses of Shanchol^TM^ following a reactive OCV campaign conducted as a part of integrated cholera outbreak control interventions [54]. A total of 239 patients with acute, non-bloody diarrhea were treated at health centers in the study area. Among them, 4 died and 40 were enrolled in the primary analysis to be compared with 160 controls. Suspected cholera cases were confirmed by means of a rapid test, and controls were selected among neighbors of the same age and sex as the case patients. After adjustment for potentially confounding variables, vaccination with two complete doses was associated with 86.6% vaccine effectiveness (95% CI: 57–96, *p* = 0.001) (Table 4) [54].

During a cholera outbreak in May 2015, in Juba, South Sudan, the Ministry of Health, Medecins Sans Frontieres, and partners engaged in the first field deployment of a single dose of Shanchol^TM^ to maximize coverage during the outbreak response considering a limited vaccine supply [55]. The mass vaccination campaign lasted from July to August 2015 and additional groups were targeted from August to September 2015, including neighbors of cholera cases, a military camp, prisoners, and health-care workers. Eventually, 165,000 people were vaccinated with a single dose of oral cholera vaccine. The short-term vaccine effectiveness of a single dose of Shanchol™ in a cholera outbreak setting was assessed by a case-cohort approach, using information on vaccination status and disease outcomes from a random cohort recruited from Juba and with all cholera cases confirmed by PCR for *V. cholerae* O1. The unadjusted single-dose vaccine effectiveness was 80.2% (95% CI: 61.5–100.0) and after adjusting for potential confounders was 87.3% (95% CI: 70.2–100.0) over a 2 month period (Table 4) [55,56].

In a known hotspot in and around Lake Chilwa in Malawi, a cholera outbreak started in December 2015 mostly affecting mobile, difficult-to-reach fishermen who settle in floating huts on the lake. A novel vaccine distribution strategy was deployed in early 2016 where fishermen were given the first vaccine dose under supervision, and the second dose in a sealed bag to be taken two weeks later [57]. Patients with diarrhea were admitted to health facilities near the lake and a stool sample was collected for PCR testing [57]. Using a case-control test-negative design where cases (PCR-positive for *V. cholerae* O1) were compared against controls (patients with diarrhea but PCR-negative), and comparing the proportions of vaccinated among cholera cases versus the general fishermen population, vaccine effectiveness was assessed [57]. Among 145 participants, 120 were fishermen living on the lake. Vaccine effectiveness at 3 months was 90.0% (95% CI: 38.8–98.4) among fishermen living on the lake, and 83.3% (95% CI: 20.8–96.5) among all participants in the case-control test-negative design (Table 4) [57]. A cost-effectiveness analysis of this reactive OCV vaccination campaign indicated it was a cost-effective intervention, relative to the Malawi gross domestic product per capita [58].

Another single dose effectiveness study was conducted in Lusaka, Zambia. A matched case-control, test-negative case-control and case-cohort design was adopted [59]. In all three study designs, cases were defined as culture confirmed cholera cases. From April to June 2016, 211 suspected cases (66 confirmed cholera cases and 145 non-cholera diarrhea cases), 1055 matched controls and a cohort of 921 were recruited [59]. Adjusted vaccine effectiveness of one dose of Shanchol^TM^ was 88.9% (95% CI: 42.7–97.8) in the matched case-control study, 80.2% (95% CI: 16.9–95.3) in the test-negative case-control design and 89.4% (95% CI: 64.6–96.9) in the case-cohort study (Table 4) [59]. All three study designs confirmed high protection of OCV against medically attended cholera infection for at least 2 months after immunization [59]. The studies in Juba, Sudan, and Zambia established the immediate population level impact of this practical outbreak response approach. In addition, a cost-effectiveness analysis of the single dose OCV reactive campaign in densely populated areas of Lusaka, Zambia showed that the single dose vaccine approach was cost-effective [60].

##### Effectiveness Studies in Endemic Areas for a Preventive Intervention

Shanchol^TM^ was administered during a mass vaccination campaign from May to June 2011 in a rural cholera-endemic area of Puri District, Odisha State, India [61]. Of 51,488 eligible residents, 31,552 individuals received at least one dose and 23,751 residents received two doses of the vaccine. An effectiveness study was conducted using a test-negative, case-control design. Controls were patients seeking treatment for *V. cholerae* negative diarrhea. Over the two years, residents seeking care for diarrhea at one of five designated health facilities were asked to enroll in the study. At the end of two years, the adjusted protective effectiveness for persons receiving two doses was 69.0% (95% CI: 14.5–88.8) (Table 5) [61]. In addition, using a cholera vaccination economic model, OCV was found to be very cost-effective in high risk populations in India based on WHO criteria [62].

A cluster-randomized, open-label effectiveness trial was conducted in Dhaka, Bangladesh. The aim of this study was to assess overall protection conferred by a two-dose regimen of Shanchol^TM^ or OCV in combination with WASH (Water, Sanitation and Hygiene) related behavioral change against hospital admission for severely dehydrating cholera during two years of follow-up. Clusters were geographically assigned with buffer zone of at least 30 m between clusters to minimize spillover of the behavioral intervention to clusters not assigned to this intervention. Through routine government immunization services, the first dose was provided in 2011, and the second dose was given between March and April 2011 [63]. Of 268,896 people present at baseline, 267,270 were analyzed: 94,675 assigned to vaccination only, 92,539 assigned to vaccination and WASH intervention, and 80,056 assigned to non-intervention, through randomization to 90 geographical clusters to one of three groups (1:1:1). Vaccine coverage was similar between the intervention groups: 65% in the vaccination only group and 66% in the vaccination and WASH intervention group. The primary outcome was overall protective effectiveness of the vaccine against severely dehydrating cholera, with severe dehydration. Among 528 cholera episodes detected, 226 (43%) were severely dehydrated; 65 in the vaccination only group, 55 in the vaccination and behavioral change group, and 106 in the non-intervention group, for the analysis of overall protection. During the 2 year follow up, overall vaccine effectiveness was 37% (95% CI, lower bound 18%, *p* = 0.002) in the vaccination group and 45% (95% CI, lower bound 24%, *p* = 0.001) in the vaccination and behavioral change group (Table 5). The results show that even with moderate vaccine coverage, the incidence of severely dehydrating cholera was reduced by OCV in the study population, irrespective of vaccination status, when vaccine was administered via routine government services in a densely populated urban setting [63].

The participants were further followed for 4 years until 2015. Overall OCV protection was 36% (95% CI: 19–49) (Table 5). Cumulative total vaccine protection was notably lower in the age below 5 years old (24%; 95% CI: −30–56) compared to the 5 years old and above (49%; 95% CI: 35–60), although the differences in protection for the two age groups were not significant (*p* = 0.3308) [64]. In this study, the protective effectiveness was lower than that in the Kolkata trial using the same vaccine. In the Kolkata study, 9% of the participants migrated out of the study area during the 2 years of follow-up compared with 58% in the Dhaka study. Comparison between pre-migration cholera rates among those who migrated out versus rates among those who did not, gave no indication that outmigration directly affected vaccine protection against cholera. However, a high rate of migration could have two effects that could have decreased vaccine protection: first, influx of non-vaccinees into vaccinated clusters could have diluted vaccine coverage and the indirect effect; and second, migration of vaccinees into non-intervention clusters could have contaminated the control group. Therefore, the authors suggested that the estimated vaccine protection is conservative compared with a mass vaccination program in a large geographic population, within which most migrations would occur [63].

### 3.3. Euvichol^®^/Euvichol-Plus

In 2010, IVI initiated a partnership with EuBiologics Co., Ltd. (Seoul, Korea) to conduct a technology transfer of OCV and establish an additional manufacturer to meet the increasing global demand [7]. Because the manufacturing process and composition of Euvichol^®^ was identical to Shanchol^TM^, the product was licensed and subsequently WHO prequalified based on an immune non-inferiority study in 2016 [7]. To further improve Euvichol^®^, EuBiologics changed the presentation of the vaccine from conventional glass vials to plastic tubes (Euvichol-Plus, thimerosal free), to facilitate delivery in emergency situations or humanitarian campaigns [7]. Consequently, no direct efficacy study of Euvichol^®^/Euvichol-Plus has been conducted and it is assumed to have the same performance characteristics of Shanchol^TM^. Since the creation of the stockpile more than 100 million doses of OCV have been shipped in response to requests from more than 20 countries [65]. Currently, EuBiologics is the main supplier to the global stockpile and Euvichol-Plus is the primary product in use as effectiveness of OCV in real world settings continues to be explored.

## 4. Live Attenuated Monovalent (O1) Vaccines: Orochol^®^/Mutacol^®^/Orochol-E^®^, Vaxchora^®^

Human challenge studies provided evidence that infection with virulent *V. cholerae* resulted in robust immune responses including secretory IgA, and subsequent protection against re-challenge as long as three years later [66,67]. Consequently, researchers at the University of Maryland Center for Vaccine Development pursued a live attenuated vaccine approach. CVD 103-HgR is a *V. cholerae* serogroup O1, serotype Inaba, classical biotype strain (569B) which has been genetically modified to remove the gene encoding the cholera toxin A subunit (the toxic subunit), and contain an insertion of a Hg++ resistance gene to enable differentiation of the vaccine strain from wild type. The initial recombinant CVD 103-HgR commercial formulation was manufactured by the Swiss Serum and Vaccine Institute which was renamed to Berne Biotech Ltd. (acquired by Crucell in 2004 and Crucell was acquired by Johnson & Johnson in 2011) (Bern, Switzerland) [68]. A formulation containing lyophilized ~5 × 10^8^ CFU per dose of *V.cholerae* CVD 103-HgR contained in a capsule was commercialized under the trade names Orochol^®^ (licensed in Switzerland, Australia, New Zealand, and a number of other countries) and Mutacol^®^ (licensed in Canada) [69].

CVD 103-HgR was first evaluated in four experimental cholera challenge studies (CVD 9003, 9007, 19002, 45000) of five US cohorts encompassing 64 vaccinees and 47 controls who were challenged with either El Tor Inaba N16961 (1 month or 3 months postvaccination), El Tor Ogawa E7946 (1 month postvaccination), or El Tor Ogawa 3008 (10 days or 1 month postvaccination) between 1987 and 1999 [69]. The combined results showed 92.7%, 95.4%, 79.0% and 67.6% of protective efficacy for severe diarrhea for ≥5 L diarrheal stool, ≥3 L diarrheal stool, ≥1 L diarrheal stool and any diarrhea, respectively (Table 6) [69,70].

A pre-licensure field study was conducted in North Jakarta, Indonesia between 1993 and 1997. A randomized, double-blind, placebo-controlled efficacy trial of one dose of CVD 103-HgR was performed in 67,508 persons aged 2–41 years. Participants ingested vaccine or placebo and were followed for four years using hospital-based surveillance. Cholera incidence was lower than expected during the study surveillance period and 103 cases of *V. cholerae* O1 El Tor diarrhea were detected, 93 evaluable for vaccine efficacy (43 vaccine, 50 placebo; efficacy = 14% (lower single-tailed 95% CI: −24) (Table 6) [71]. Considering the discrepancy between the results of the human challenge experiments in North American volunteers and participants in Indonesia, it was hypothesized that a higher antigen exposure might be required for use in endemic settings. Consequently, a high-dose formulation containing ~5 × 10^9^ CFU per dose was commercialized as Orochol-E^®^ for use in low and middle income setting at risk for cholera.

In Pohnpei Island, Micronesia, an effectiveness study using mass vaccination with the single-dose Orochol-E^®^ was performed. As a response to a cholera outbreak in 2000, the Federated State of Micronesia requested vaccine, and WHO supported delivery of 48,000 doses of Orochol-E^®^ which had been donated by the manufacturer (Berna Biotech, Bern, Switzerland). The vast majority of vaccinated individuals living in Pohnpei Proper received Orochol-E^®^ between 9 and 19 September 2001. Vaccination was organized between 18 and 23 September 2001 on Pohnpei outer islands. The vaccine effectiveness was retrospectively evaluated as an adjunct reactive measure during a cholera epidemic. The crude vaccine effectiveness estimated was 79.2% (95%CI: 71.9–84.6) in the target population (Table 6). However, as the study was conducted retrospectively some have identified possible shortcomings of the study including potential biases and confounding factors that may have influenced the outcome [72].

Berna Biotech was acquired by Crucell which discontinued production of Orochol^®^ and Orochol-E^®^ and the license to the intellectual property reverted to the University of Maryland, Baltimore. In 2009, PaxVax (acquired by Emergent in 2018), licensed rights to again commercialize CVD 103-HgR from the University of Maryland (College Park, MD, USA) with the goal of obtaining FDA approval as a travelers vaccine. The Paxvax product, Vaxchora^®,^ contains the identical strain with the same phenotypic and genomic properties as Orochol^®^ at 2 × 10^8^ CFU or more [69].

A randomized, placebo-controlled human challenge study of Vaxchora^®^ was conducted to evaluate the vaccine efficacy. The phase 3 trial enrolled 197 healthy adult participants aged 18–45 years old in academic clinical research facilities in three US sites. Participants were randomly allocated to receive a blinded product, single dose of the vaccine (*n* = 95) or saline placebo (*n* = 102). The primary endpoint, moderate (≥3 L diarrheal stool) to severe (≥5 L diarrheal stool) diarrhea, occurred in 39 of 66 (59.1%) placebo controls but only 2 of 35 (5.7%) vaccinees at 10 days (vaccine efficacy, 90.3%; *p* < 0.0001) and 4 of 33 (12.1%) vaccinees at 3 months (vaccine efficacy, 79.5%; *p* < 0.0001) (Table 6) [73].

Currently, Vaxchora^®^ is approved by the US FDA for use in persons 2 through 64 years of age traveling to cholera-affected areas. It is supplied as two packets with one containing buffer and the other the live agent both of which must be reconstituted in water prior to administration. The higher cost, complexity of administration, and the cold chain requirements (−25 to −15 °C) limit its utility beyond the traveler’s market. Further, the usefulness of CVD 103-HgR in controlling cholera in endemic countries remains unclear given the disparate results observed in Indonesia and Micronesia [74].

## 5. Discussion

Because of the size and expense of conducting clinical trials against cholera, randomized and placebo controlled Human challenge studies have played an incredibly important role in the development of oral cholera vaccines. Although inherently artificial in setting, indication of potential efficacy in a human challenge has been the threshold for moving candidate vaccines to large trials in at risk populations. Across numerous efficacy and effectiveness studies of multiple formulation variations including killed and live whole cell oral vaccines, it is clear that oral cholera vaccines have the potential for high impact and their use can be adapted to numerous settings.

The intense and structured process required for end point evaluation in a randomized controlled trial is necessary to develop convincing data for registration, and also enabled identification of additional important vaccine characteristics such as the cross protection of Dukoral^®^ to a different enteric pathogen LT-ETEC, and the time to onset of protection. We can look at the totality of the experience with the WC-OCVs, whether with or without CTB, and see that the Pampas study was an outlier with the negligible vaccine efficacy in the first year. It is important and cautionary to recognize that even a randomized controlled trial can give misleading results. Effectiveness studies of WC-BS and WC vaccines also have outliers, but the tendency seen is for effectiveness of the vaccine used in public health settings to be somewhat higher than estimated in randomized controlled trials. Effectiveness studies of OCV targeting substantial proportions of the population as public health campaigns were able to demonstrate the indirect or “herd effect” of vaccination likely from reduced transmission of cholera in the community. The reduced transmission may elevate the individual protection of vaccinated participants and is shared by unvaccinated neighbors. From a public health perspective, the additional protection of unvaccinated neighbors is a significant attribute typically only measurable in an effectiveness study.

Because effectiveness studies seek to include the heterogeneity of real-world populations, they provide the opportunity to understand vaccination effects on special populations often excluded from randomized clinical trial, such as pregnant women, those with chronic diseases, or infected with HIV. In the case of OCV, the observational study of vaccinated individuals in a public health campaign provided the first reassuring information about safety of the vaccine in pregnant women and in a community with high HIV seroprevalence. Further, government level policymakers and finance ministers want to understand the cost-effectiveness of vaccines which requires data generated by implementation of vaccines in a public health setting.

Randomized controlled trials are the gold standard for regulators to reduce bias. Although various effectiveness study designs are implemented to adjust for bias, it needs to be acknowledged that, in observational studies of public health use of vaccines, there may be important differences between those vaccinated and unvaccinated. These two groups have had their status selected by choice or circumstance. Their individual characteristics have not been randomly distributed between groups and in spite of bias adjustments, the two groups may differ in ways that impact the outcome of vaccination.

Efficacy and Effectiveness studies consistently demonstrate that OCVs prevent cholera, and both study types contribute important information about the performance characteristics of a vaccine and the impact that they will have when implemented in real-world populations at risk for disease.

## Figures and Tables

**Table 1:** Composition of killed whole-cell OCV.

WC	Strain	Serotype	Biotype	Inactivation	WC Monovalent Vaccine with or without CTB							WC Bivalent Vaccine		
					Black 1987 Challenge Study	Clemens 1986 Matlab, Bangladesh		Sanchez 1994 Military Recruits, Peru	Taylor 2000 Pampas, Peru	Dukoral^®^	Hue, Vietnam			mORC-VAX^TM^ Shanchol^TM^ Euvichol^®^/ Euvichol-Plus
											Field Trial 1992	Campaign 1998	Campaign 2000	
**O1**	**Cairo 48**	**Inaba**	**Classical**	**Heat**	5 × 10^10^	2.5 × 10^10^	2.5 × 10^10^	2.5 × 10^10^	2.5 × 10^10^	3.125 × 10^10^	2.5 × 10^10^	2.5 × 10^10^	-	300
	**Cairo 50**	**Ogawa**	**Classical**	**Heat**	5 × 10^10^	2.5 × 10^10^	2.5 × 10^10^	2.5 × 10^10^	2.5 × 10^10^	3.125 × 10^10^	2.5 × 10^10^	2.5 × 10^10^	2.5 × 10^10^	300
	**Cairo 50**	**Ogawa**	**Classical**	**Formalin**	-	2.5 × 10^10^	2.5 × 10^10^	2.5 × 10^10^	2.5 × 10^10^	3.125 × 10^10^	-	-	-	300
	**Phil 6793**	**Inaba**	**El Tor**	**Formalin**	1 × 10^11^	2.5 × 10^10^	2.5 × 10^10^	2.5 × 10^10^	2.5 × 10^10^	3.125 × 10^10^	2.5 × 10^10^	2.5 × 10^10^	2.5 × 10^10^	600
	**569B**	**Inaba**	**Classical**	**Formalin**	-	-	-	-	-	-	2.5 × 10^10^	2.5 × 10^10^	2.5 × 10^10^	-
**O139**	**4260B**	**-**	**-**	**Formalin**	-	-	-	-	-	-	-	5.0 × 10^10^	5.0 × 10^10^	600
**Total WC**					2.0 × 10^11^	1.0 × 10^11^	1.0 × 10^11^	1.0 × 10^11^	1.0 × 10^11^	1.25 × 10^11^	1.0 × 10^11^	1.5 × 10^11^	1.25 × 10^11^	2100
**CTB**	**Purified CTB**				5 mg	1 mg	-	-	-	-	-	-	-	-
	**Recombinant CTB**				-	-	-	1 mg	1 mg	1 mg	-	-	-	-

Units for whole-cells: cells (bacterial count), ELISA Units (EU) of lipopolysaccharide (LPS); WC; Whole-cell, CTB; Cholera Toxin B subunit.

**Table 2:** Efficacy and effectiveness of WC-CTB.

Efficacy	Challenge Study 1 Month	Matlab, Bangladesh 12 Months *	Military Rrecruits, Peru 5 Months	Pampas, Peru 12 Months
WC-CTB	64%	62%	86%	0%
WC	56%	58%	NA	
**Effectiveness**	**Beira, Mozambique** **5 Months**	**Zanzibar, Tanzania** **15 Month**		
WC-CTB	84%	79%		

* Study assesses vaccine efficacy in children 2–15 years of age and women older than 15 years.

**Table 3:** Efficacy of Shanchol.

Area	Kolkata, India			Dhaka, Bangladesh	
Number of Doses	2			1	
**Follow** **Up** **Duration**	2 years	3 years	5 years	6 months	2 years
**Efficacy**	67%	66%	65%	40%	39%

Efficacy for 1 year or older.

**Table 4:** Effectiveness of Shanchol^TM^ in outbreak setting.

Area	Haiti			Guinea	Juba, Sudan	Lake Chilwa, Malawi	Lusaka, Zambia
	Port-Au-Prince	Bocozel and Grand Saline					
**Number of Doses**	1 or 2	1 or 2	2	2	1	1 or 2	1
**Follow** **up** **Duration** **Effectiveness**	37 M	4–24 M	4 Y	6 M	2 M	3 M	2 M
	97.5%	63%, 58% ^a^	76%	86.6%	87.3%	90.0%	88.9% ^b^

^a^ VE was 63% (95% CI: 8–85) by self-reported vaccination and 58% (95% CI: 13–80) for verified vaccination. ^b^ 88.9% (95% CI: 42.7–97.8) in the matched case-control study, 80.2% (95% CI: 16.9–95.3) in the test-negative case-control design and 89.4% (95% CI: 64.6–96.9) in the case-cohort study. M; months, Y; years.

**Table 5:** Effectiveness of Shanchol^TM^ in endemic areas.

Area.	Puri District, Odisha State, India	Dhaka, Bangladesh	
Number of Doses	2	2	
Follow up Duration	2 years	2 years	4 years
Effectiveness	69.0%	37%	36%

**Table 6:** Efficacy and effectiveness of live attenuated OCV.

Name of Live Attenuated OCV	Orochol^®^/ Mutacol	Orochol-E^®^	Vaxchora^®^		Orochol-E^®^
**Composition**	2 to 10 × 10^8^ CFU [75]	~5 × 10^9^ CFU	4 × 10^8^ to 2 × 10^9^		~5 × 10^9^ CFU
		**Efficacy**			**Effectiveness**
**Area**	**Challenge study**	**North Jakarta,** **Indonesia**	**Challenge study**		**Pohnpei Island,** **Micronesia**
**Follow up Duration**	10 D, 1 M, 3 M	4 Y	10 D	3 M	3 M
**Effectiveness**	67.6% ^a^	14%	90.3%	79.5%	79.2%

^a^ The combined results in 4 challenge studies showed 92.7%, 95.4%, 79.0%, and 67.6% of protective efficacy for severe diarrhea for ≥5 L diarrheal stool, ≥3 L diarrheal stool, ≥1 L diarrheal stool and any diarrhea, respectively. D; days, M; months, Y; years.
